# Live long and PROSPERO: A comment on Chiu and coworkers

**DOI:** 10.1111/jch.14358

**Published:** 2021-08-29

**Authors:** Phillip J. Tully, Rianne A.A. de Heus, The VARIABLE BRAIN Consortium

**Affiliations:** ^1^ Freemasons Centre for Male Health and Wellbeing Adelaide Medical School The University of Adelaide Adelaide Australia; ^2^ Department of Geriatric Medicine Radboudumc Alzheimer Center Radboud University Medical Center Donders Institute for Brain Cognition and Behavior Nijmegen The Netherlands

Dear Editor,

The *a priori* registration of systematic reviews provides a way to describe the methods for a meta‐analysis before Review Paper selection commences. Chiu and coworkers^1^ report a systematic review of blood pressure variability, deviating from the protocol registered on PROSPERO (CRD42020190429).^2^ We raise concern relating to the lack of transparency in methods^1^ against the PROSPERO registration (CRD42020190429).^2^ The registered review question was *“What is the difference between long‐term and short‐term blood pressure variability (BPV) in relation with cognitive decline or incidence of dementia regarding general population or specific subgroup?* ^2^ *”* Concerningly, the discrepancy between the review's main aim and that of the published version^1^ was not addressed, nor was a PRISMA checklist^3^ provided with this paper,^1^ which specifies for authors to describe any changes from the protocol (item 24c of PRISMA checklist^3^). Moreover, in the published version,^1^ it is clear the meta‐analysis was unable to sufficiently answer the review question,^2^ with only one study identified for mid‐term BPV, and three or less studies analyzed for long‐term BPV. Other limitations remain. For example, the review includes studies with stroke or trans ischemic attack who are prone to sudden onset cognitive impairment, versus the more insidious onset in dementia. Also, the title implies longitudinal cohort studies were included, when in fact most of the contributing data was obtained retrospectively from a primary care database registry, which may introduce self‐selection and other biases.

Other advantages from *a priori* registration of systematic reviews include reducing redundancy and wasted resources allocated to overlapping systematic reviews.^4^ On the 21^st^ of May 2021, at our request, PROSPERO sought clarification from Chiu and coworkers^1^ how their protocol was different from The VARIability in BLood pressurE and BRAIN outcomes consortium's (VARIABLE BRAIN) registered review CRD42017081977^5^ and peer reviewed protocol.^6^ This is especially important because one limitation reported by Chiu and coworkers^1^ is that *“the BP level may still have a greater influence than true variability.”* We have shown that this is not the case in older adults. Specifically, in a meta‐analysis of 54 effect sizes, a direct comparison showed that mean BP effect sizes were less strong than BPV effect sizes (*p* < .01), suggesting that the relative contribution of BPV exceeded that of mean BP for dementia and cognitive impairment risk. This raises the possibility that BPV is a novel marker for neurodegeneration in older adults, whereas in mid‐life, hypertension and elevated mean blood pressure remain as prominent modifiable risk factors.^7^, ^8^

## CONFLICT OF INTEREST

The authors have no competing interests.
